# Cross-linked cyclodextrin-based material for treatment of metals and organic substances present in industrial discharge waters

**DOI:** 10.3762/bjoc.12.172

**Published:** 2016-08-12

**Authors:** Élise Euvrard, Nadia Morin-Crini, Coline Druart, Justine Bugnet, Bernard Martel, Cesare Cosentino, Virginie Moutarlier, Grégorio Crini

**Affiliations:** 1 Chrono-environnement, UMR 6249 usc INRA, University of Bourgogne Franche-Comté, 16 route de Gray, 25000 Besançon, France; 2 UMET UMR 8207, Ingénierie des Systèmes Polymères, University of Lille 1, 59655 Villeneuve d’Ascq, France; 3 G. Ronzoni Institute for Chemical and Biochemical Research, 81 via G. Colombo, 20133 Milano, Italy; 4 Chrono-environnement, Institut UTINAM, UMR 6213, University of Bourgogne Franche-Comté, 16 route de Gray, 25000 Besançon, France

**Keywords:** alkylphenols, adsorption, cyclodextrin, metals, polycyclic aromatic hydrocarbons

## Abstract

In this study, a polymer, prepared by crosslinking cyclodextrin (CD) by means of a polycarboxylic acid, was used for the removal of pollutants from spiked solutions and discharge waters from the surface treatment industry. In spiked solutions containing five metals, sixteen polycyclic aromatic hydrocarbons (PAH) and three alkylphenols (AP), the material exhibited high adsorption capacities: >99% of Co^2+^, Ni^2+^ and Zn^2+^ were removed, between 65 and 82% of the PAHs, as well as 69 to 90% of the APs. Due to the structure of the polymer and its specific characteristics, such as the presence of carboxylic groups and CD cavities, the adsorption mechanism involves four main interactions: ion exchange, electrostatic interactions and precipitation for metal removal, and inclusion complexes for organics removal. In industrial discharge waters, competition effects appeared, especially because of the presence of calcium at high concentrations, which competed with other pollutants for the adsorption sites of the adsorbent.

## Introduction

Although considerable efforts have been made by the industrial sector over the last 30 years, the problem of water pollution still remains a significant concern. Particularly affected by this issue are the discharge waters (DWs) of the surface treatment (ST) industries known for using large amounts of water and chemicals in their manufacturing processes. Despite these industries have their own treatment plants, generally physicochemical decontamination steps, the DWs still contain non-negligible amounts of pollutants. Among them, metals (in particular chromium, nickel and zinc) are commonly found at concentrations in the range of milligrams per liter, and organic molecules, such as polycyclic aromatic hydrocarbons (PAHs) and alkylphenols (APs) at concentrations varying from hundreds of nanograms per liter to some micrograms per liter [1].

However, it is extremely difficult to remove pollutants present at low concentrations (a few hundreds of micrograms per liter for some organic substances in DWs). For this purpose, specific systems can be added, called effluent finishing treatments. A sequential dual approach can be considered: firstly, adsorption onto carbon to remove organics (e.g., solvents, oils, PAHs and volatile organic compounds) combined with ion-exchange and/or chelation by means of organic resins to remove inorganic pollutants (e.g., metals and anions such as fluorides). Charles et al. [2] recently reported that this type of sequence is acknowledged for its efficiency. However, it is an approach to water treatment that combines two methods of separation using two distinct commercial materials. To our knowledge, materials able to combine the two functions are rare. Recently, bifunctional natural derivatives have been proposed for this purpose. For example, Zhao et al. [3] proposed a new cyclodextrin-based material for the simultaneous adsorption of metals and cationic dyes. Zhang et al. [4] studied the removal of cobalt and 1-naphthol onto magnetic nanoparticles containing cyclodextrin and iron and Yang et al. [5] proposed a new nanocomposite adsorbent for the simultaneous removal of organic and inorganic substances from water.

Here, we propose to use a single cross-linked cyclodextrin-based polymer for the removal of metals and organic pollutants present in polycontaminated effluents. Cyclodextrins (CDs), synthetic substances obtained from the enzymatic degradation of starch, belong to the family of cage molecules. They present remarkable encapsulation properties leading to a host–guest relationship with organic substances [6–9]. These cyclic oligosaccharides are water soluble in their native form and are often modified to prepare novel insoluble CD-based materials. Two patents published by Martel et al. [10], and Trotta et al. [11] can be consulted for the use of carboxylic acids and pyromellitic dianhydride, respectively, as agents to cross-linking CDs. Other cross-linking agents such as epichlorohydrin, ethylene glycol diglycidyl ether, glutaraldehyde, benzoquinone or isocyanates can be also used [1,12].

The main objective of the study was to investigate the adsorption capacities of a non-conventional and versatile CD-based material crosslinked with 1,2,3,4-butanetetracarboxylic acid (BTCA) toward several inorganic and organic elements. Performances of such systems were evaluated in the presence of spiked solutions and real DWs from ST industry containing five metals, 16 PAHs, three APs (model pollutants present in DWs from treatment-surface industries) in the presence and in the absence of calcium. The polymer showed high adsorption capacities in spiked solutions but adsorption strongly decreased in discharge waters due to some competition effects, notably between inorganics for adsorption sites.

## Results

### Material characterization

**Figure 1:**
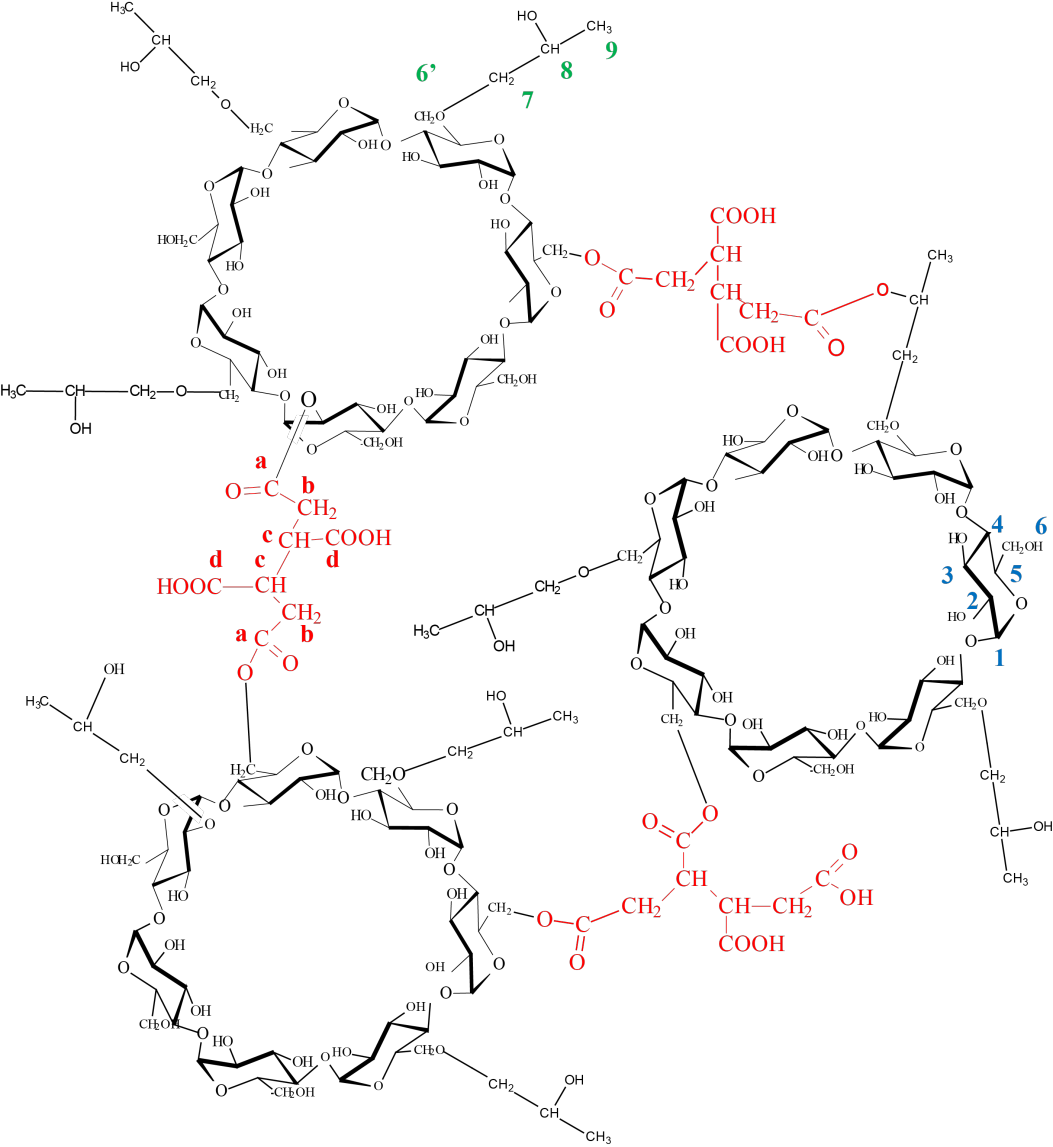
Chemical structure of the non-activated polyBTCA-CD.

The cross-linked polymer used in this study is presented in Figure 1. In the control test (solution without pollutant), polymer addition led to large pH variations. The non-activated polyBTCA-CD (COOH form) decreased the pH value from 6 to 4.3 after 20 min of shaking whereas activated polyBTCA-CD (COO^−^Na^+^ form) led to a pH increase from 6 to 7.2 after 5 min shaking. In both cases the final pH remained constant over several hours.

**Figure 2:**
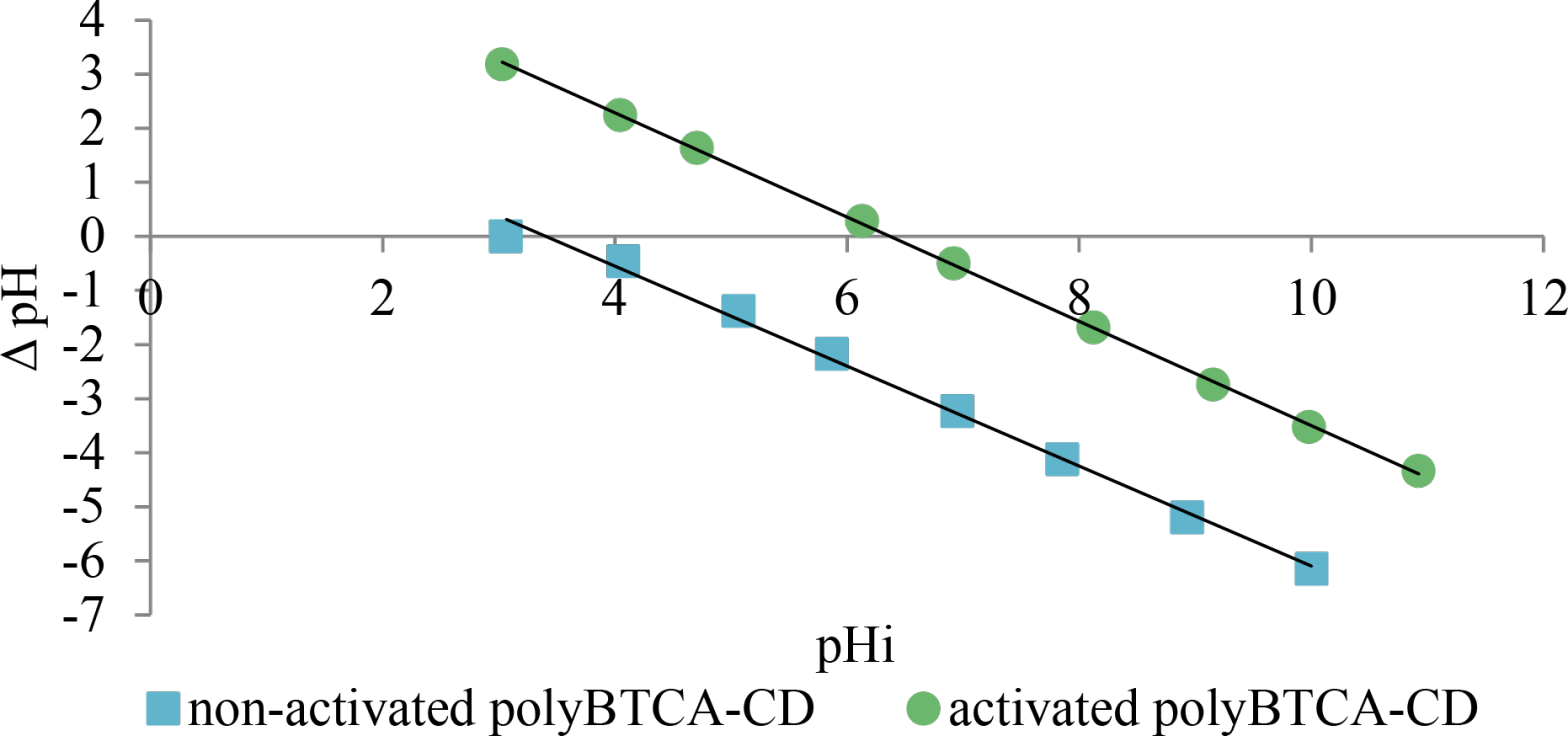
Determination of the PZC for the non-activated and activated polyBTCA-CD polymers (pHi: initial pH value, see also Experimental section).

The ion-exchange capacity (IEC) was equal to 0.705 mmol of COOH functions per gram of polymer. The point of zero charge (PZC) was plotted and followed the linear equations y = −0.9639*x* + 6.1422 and y = −0.9233*x* + 3.138 indicating a pH of 6.4 and 3.4 for the PZC of activated and non-activated polyBTCA-CD, respectively (Figure 2).

**Figure 3:**
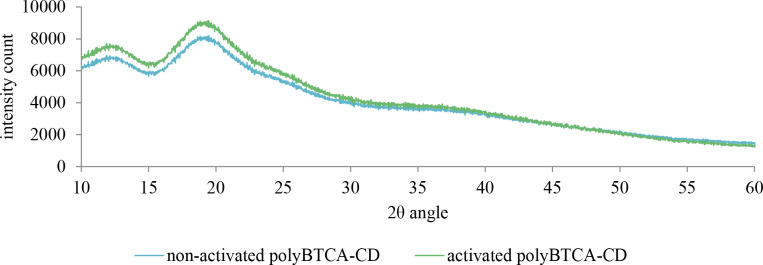
XRD pattern of the two polymers: non-activated and activated polyBTCA-CD.

Figure 3 shows the XRD pattern of non-activated and activated polymers. These diffractograms indicate a wide amorphous peak between values of 2θ of 10 and 30°, highlighting that polymers are amorphous. No significant differences were observed between the two types of polymer.

**Figure 4:**
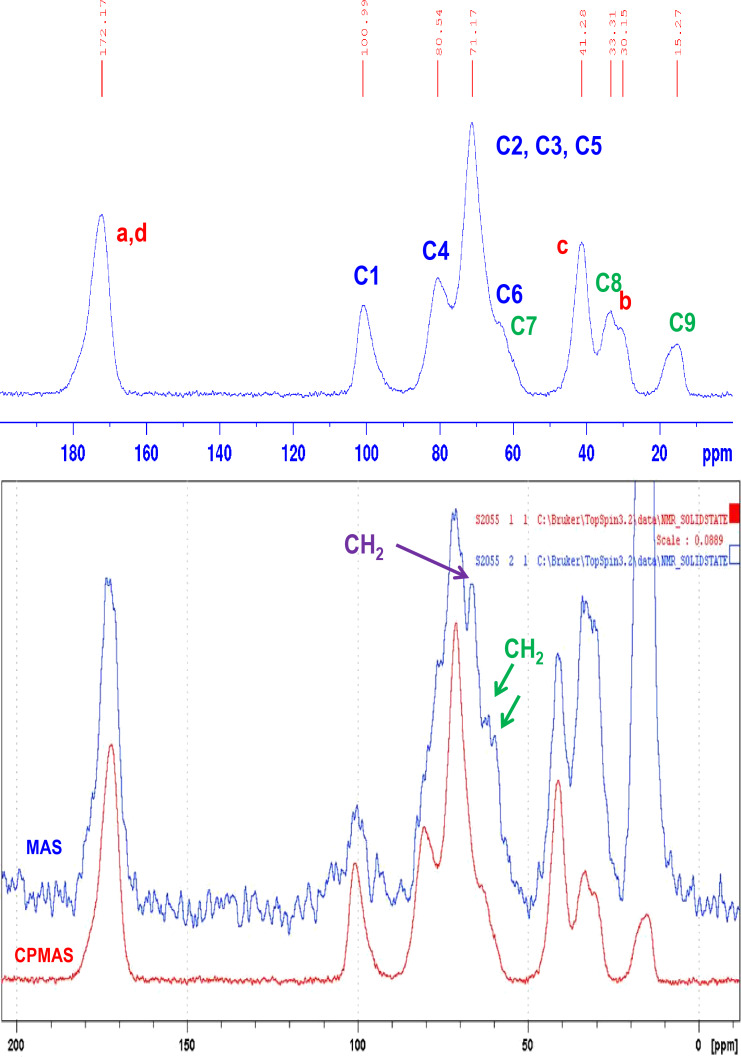
CPMAS and MAS spectra of polyBTCA-CD.

Solid-state ^13^C NMR spectra of the polymer are presented in Figure 4. The cross polarization magic angle spinning (CPMAS) spectrum shows the peaks of disordered cyclodextrin (broad signals) in the range of 50–110 ppm. Three strong broad bands attributable to the glucopyranose unit can be observed. The peak at 101 ppm is attributed to the anomeric carbon C-1: this confirms the presence of glucose units in the polymer. In the range of 50–110 ppm, the CH_2_ signals of CD (C-6, C-6’ and C-7) are completely hidden by the C-2, C-3 and C-5 peaks of the glucopyranose units. In the MAS spectrum, three CH_2_ signals are clear and the signals at 65, 62.1 and 60.2 ppm are attributable to CH_2_ in positions C-6’, C-6 and C-7, respectively. These attributions were confirmed using cross-polarization with a polarization inversion sequence (CPPI spectrum not shown), which revealed three CH_2_ signals due to the CD and also a peak at 30.15 ppm corresponding to CH_2_ groups introduced by the cross-linking agent. As expected, the signals of the BTCA crosslinking agent can be clearly distinguished (labeled a, b, c and d). In particular, the carbon of the carboxylic groups appears at 172.2 ppm. This peak (a,d) corresponds to esterified and free carboxylic groups of BTCA present in free carboxylic acids and in ester crosslinks, respectively. In the CPMAS spectrum we also note the presence of additional peaks due to the hydroxypropyl group present in the CD, and in particular the CH_3_ group (C-9 carbon) at 15.3 ppm. The comparison between the CPMAS and MAS spectra shows a different intensity for this methyl signal reflecting the greater mobility of this group, as expected. Paradoxically, the intensity of the carbonyl signal does not increase in the MAS spectrum compared with the signal of the methyl group of CD. Finally, no significant differences were obtained between the non-activated polyBTCA-CD and the activated polyBTCA-CD spectra.

### Metal adsorption

#### Effect of activation (NaHCO_3_ treatment)

**Figure 5:**
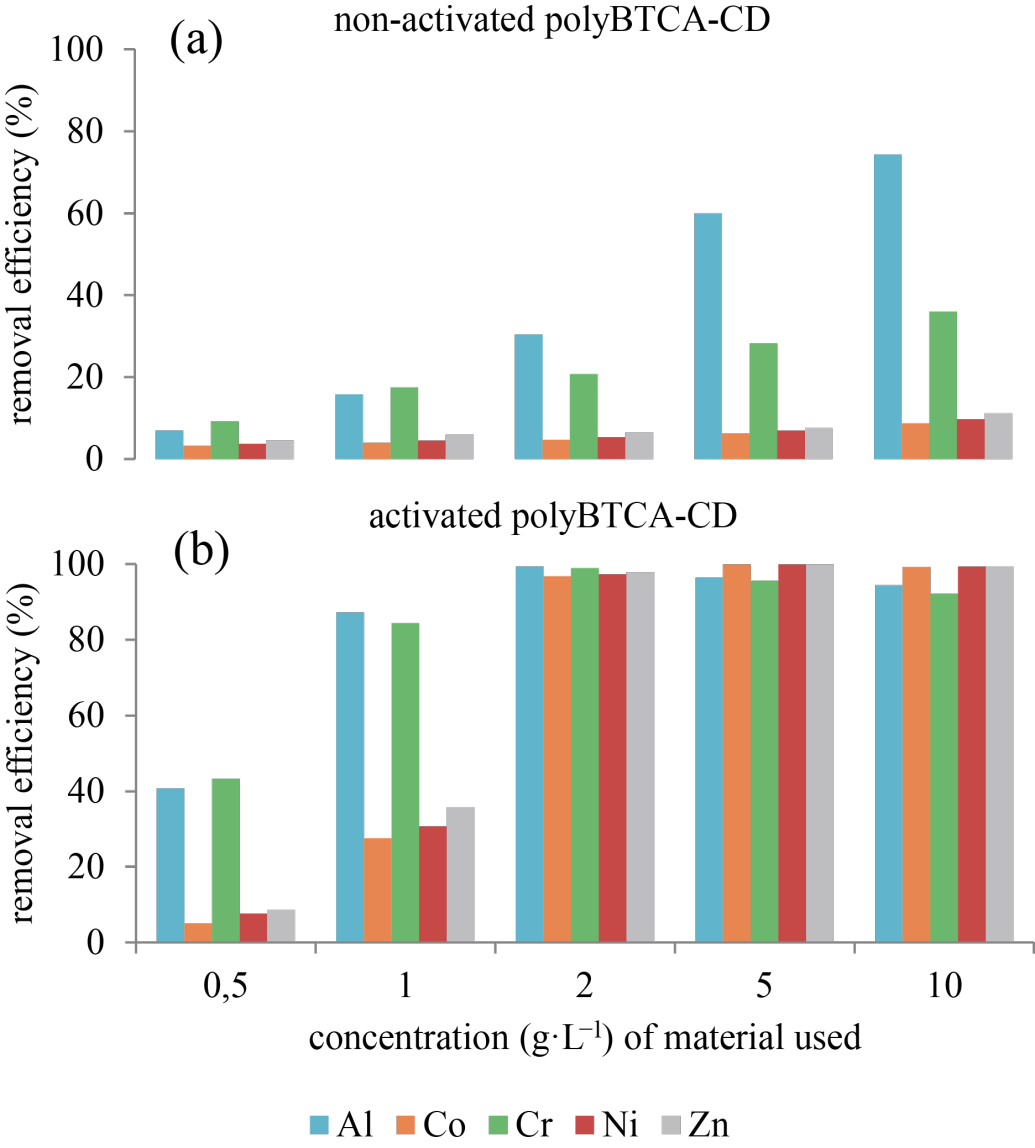
Adsorption capacity (%) of (a) the non-activated and (b) the activated (NaHCO_3_ treatment) polyBTCA-CD at different concentrations for five metals (at 10 mg·L^−1^ metal).

The activated polymer is more efficient than the non-activated polymer regardless of the dose (Figure 5). The activation enhanced the removal by 69%, 92%, 78%, 92% and 92% at a polymer concentration of 2 g·L^−1^ for Al^3+^, Co^2+^, Cr^3+^, Ni^2+^ and Zn^2+^, respectively. Moreover, it appears that a polymer concentration of 2 g·L^−1^ is enough to treat an inorganic load of 50 mg·L^−1^. Thus, for all the following experiments, the polymers were activated and a concentration of 2 g·L^−1^ was used.

#### Adsorption kinetics

**Figure 6:**
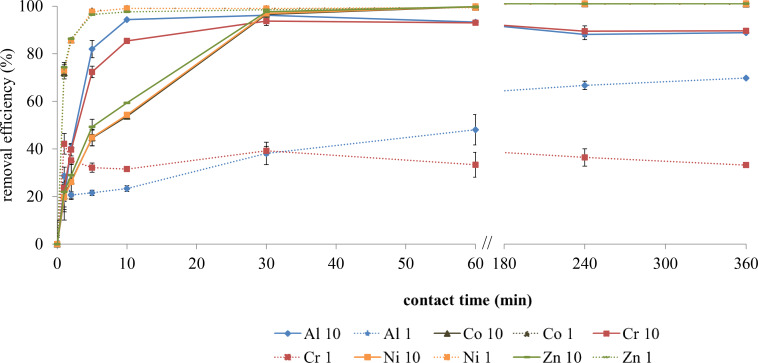
Adsorption kinetics for two solutions containing five metals at two concentrations (solution at 10 mg·L^−1^: full lines; solution at 1 mg·L^−1^: dashed lines) expressed as removal efficiency in % (*n* = 3).

Figure 6 shows the adsorption kinetics for two solutions containing five metals at 1 mg·L^−1^ and 10 mg·L^−1^. For these two concentrations, 100% removal was reached for most species except for Al1 and Cr1 systems, which reached a state of dynamic equilibrium. However, the adsorption time changed with the concentration and with the metals. At 1 mg·L^−1^ the adsorption kinetics was fast: in 5 min equilibrium was reached for the metals except for Al^3+^ (240 min). At 10 mg·L^−1^ for Co^2+^, Ni^2+^ and Zn^2+^, equilibrium time increased to 30 min, while for Al^3+^ and Cr^3+^ it decreased to 30 min. These results were obtained in triplicate with small standard deviations, indicating the reproducibility of the experiments. For the two experiments we noted an increase of pH values ranging from 3.9 and 4.4 for the initial pH (pHi) to 6.2 and 7.3 for the final pH (pHf) for concentrations of 10 mg·L^−1^ and 1 mg·L^−1^, respectively.

#### Influence of metal and calcium concentration

The results show that activated polyBTCA-CD is able to treat metal solutions from low concentrations (a few micrograms in SS3 and SS4 in Table 1) to high concentrations (ten milligrams for each metal (SS1 in Table 1). The exact composition of each spiked solution (SS) is given in the Experimental section. In addition, it was observed that without Ca^2+^ cations the removal efficiency was above 99%, except for aluminum and chromium (Table 1). When expressing these results in mmol of total metal retained per gram of polymer, it can be noted that for the highest concentration of metals (10 mg·L^−1^), the polymer retained 0.466 mmol·g^−1^. When calcium ions were added (SS5 in Table 1), Cr^3+^ was better retained than in the solution without calcium (85% compared to 38%) while the retention of Al^3+^ remained constant. For the three other metals, the retention dramatically decreased (<41%) in the presence of calcium.

**Table 1:** Efficiency of activated polyBTCA-CD expressed in % (concentration = 2 g·L^−1^) to treat several metal spiked solutions SS (*n* = 3). See Experimental section for the exact compositions of the spiked solutions SS.

	Al^3+^	Co^2+^	Cr^3+^	Ni^2+^	Zn^2+^	Ca^2+^	pHi

SS1	87 ± 4	>99	88 ± 3	>99	>99	—	6.2
SS2	66 ± 4	>99	36 ± 1	>99	>99	—	7.3
SS3	87 ± 16	>99	45 ± 7	99 ± 0	>99	—	7.3
SS4	95 ± 2	>99	38 ± 2	99 ± 0	>99	—	7.3
SS5	97 ± 0	17 ± 2	85 ± 2	22 ± 2	41 ± 7	10 ± 4	4.7

### PAH and AP adsorption

**Figure 7:**
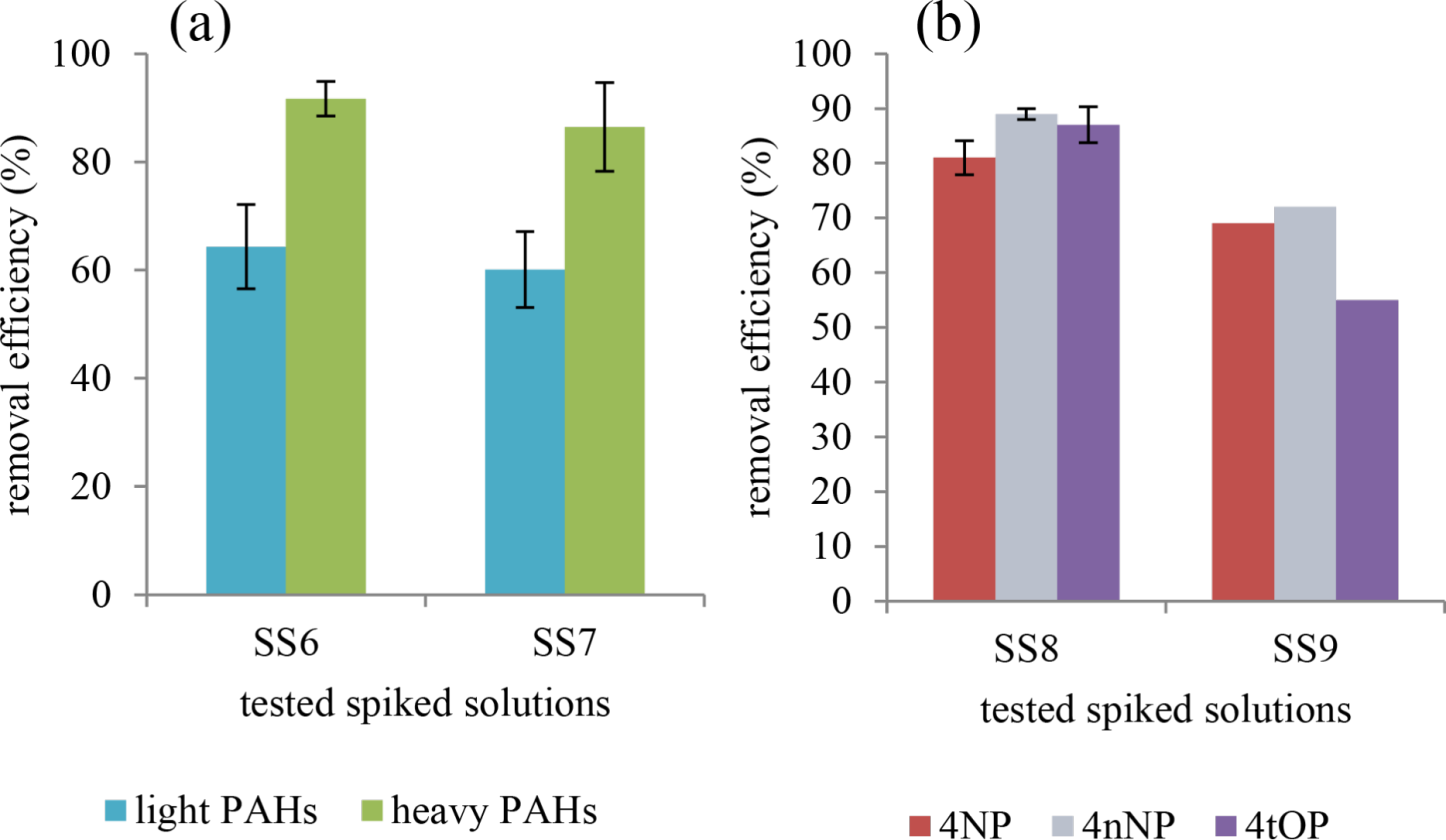
Removal efficiency (%) after treatment with activated polyBTCA-CD (concentration = 2 g·L^−1^) for (a) two concentrations of PAHs: low for SS6 and high for SS7 (*n* = 3) and (b) two concentrations of APs: low for SS8 (*n* = 3) and high for SS9 (*n* = 1) (4NP: 4-nonylphenol, 4nNP: 4-*n*-nonylphenol, 4tOP: 4-*tert*-octylphenol).

Figure 7a shows that the polyBTCA-CD can take up PAHs since the polymer removed between 74% and 79% of the global PAH load. It was also observed that removal was more efficient for heavy PAHs (89% adsorption) than for the lighter ones (62% adsorption). Following PAH adsorption the pH ranged from 6 to 7.6 at the end of the experiment. The polymer successfully removed APs as shown in Figure 7b. It can be noted that an increase of AP concentration led to a decrease in adsorption capacities, notably for 4tOP (87% to 55%; Figure 7b). For the two solutions, the pH values ranged from 6 to 8.

### Mixed metal and organic adsorption

Table 2 presents the adsorption results for the seven mixtures containing metals, PAHs, and APs. Firstly, it was noted that the removal efficiency observed in the solutions containing only one type of substance was similar to those observed in the previous experiments. The heavy PAHs were retained more than the lighter PAHs in each mixture. No adsorption difference was observed between the mixtures containing a single family of substance and those containing two or three families of substances.

**Table 2:** Removal efficiency expressed in % of metals, PAHs and APs in solutions containing either one family of substances or mixtures after treatment by polyBTCA-CD (concentration = 2 g·L^−1^, *n* = 3).

		metals	PAHs	APs	metals + PAHs	metals + APs	PAHs + APs	metals + PAHs + APs

metals	Al^3+^	66 ± 4	—	—	65 ± 14	63 ± 5	—	65 ± 5
	Co^2+^	>99	—	—	99 ± 1	99 ± 1	—	99 ± 1
	Cr^3+^	36 ± 1	—	—	28 ± 8	25 ± 13	—	24 ± 13
	Ni^2+^	>99	—	—	98 ± 2	98 ± 1	—	98 ± 1
	Zn^2+^	>99	—	—	96 ± 4	96 ± 4	—	96 ± 4
PAHs	light	—	60 ± 7	—	63 ± 3	—	62 ± 8	62 ± 4
	heavy	—	87 ± 8	—	81 ± 10	—	87 ± 3	89 ± 5
APs	4NP	—	—	80 ± 3	—	82 ± 7	80 ± 11	81 ± 1
	4nNP	—	—	89 ± 1	—	90 ± 2	85 ± 1	81 ± 2
	4tOP	—	—	83 ± 3	—	86 ± 6	86 ± 6	82 ± 4
pHf		7.3	7.6	8	7.7	7.7	7.7	7.9

### Adsorption capacities with discharge waters

#### Metal adsorption in industrial discharge waters

The polymer was then tested on DWs from a ST company that is specialized in chemical coatings and any processes for the corrosion protection of metal parts intended for the automotive and building sectors. The average concentrations of the main elements present in the industrial DW are reported in Table 3.

**Table 3:** Average concentrations expressed in mg·L^−1^ and standard errors of the main elements present in the DWs (*n* = 5).

Al^3+^	Co^2+^	Cr^3+^	Ni^2+^	Zn^2+^	Ca^2+^

1.48 ± 0.54	1.70 ± 0.74	0.04 ± 0.03	0.25 ± 0.11	0.90 ± 0.50	690 ± 156

Fe^3+^	K^+^	Mg^2+^	Mn^2+^	Sr^2+^	

0.23 ± 0.12	73.3 ± 6.56	2.84 ± 0.31	0.12 ± 0.1	0.24 ± 0.03	

**Figure 8:**
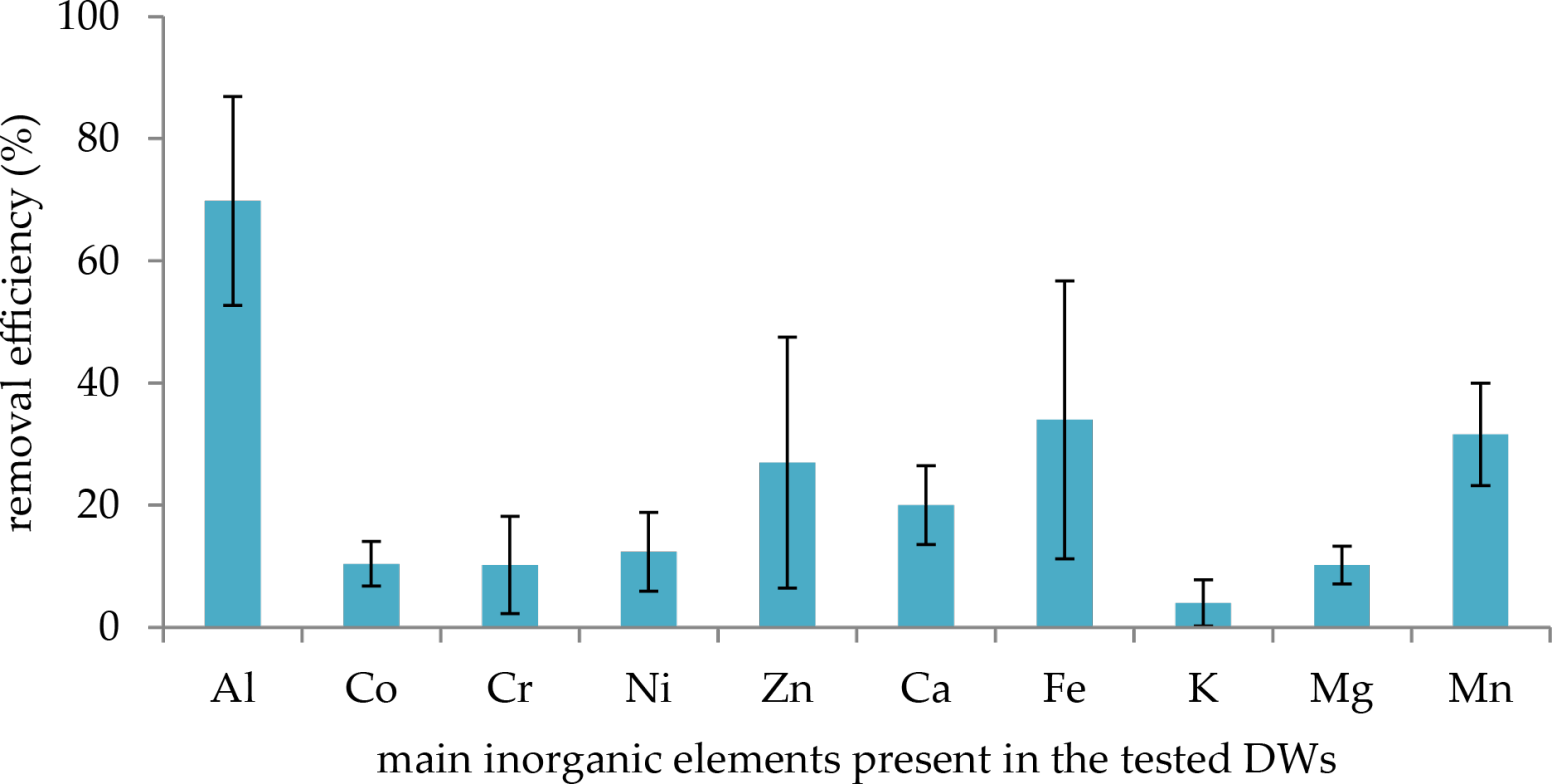
Removal efficiency (%) of inorganic elements after treatment of five DWs by polyBTCA-CD (concentrations = 2 g·L^−1^, *n* = 5).

In DWs, no pH variation was observed during the experiment (pHi = pHf = 8). The results indicate that the retention of the five metals studied above did not reach the removal efficiency obtained in SS, except for Al^3+^ (70% on average, Figure 8). Indeed, for Co^2+^, Cr^3+^, Ni^2+^ and Zn^2+^, the removal efficiencies did not exceed 30%. Nevertheless, it appears that other elements in DWs were retained by the polymer (20%, 34%, 4%, 10%, 32%, and 18% for Ca^2+^, Fe^3+^, K^+^, Mg^2+^, Mn^2+^ and Sr^2+^, respectively). Taking into account the initial concentrations of these elements (Table 3), this means that non-negligible amounts were retained. For instance, 134 mg·L^−1^ of Ca^2+^ was retained by the polymer, equivalent to 1.67 mmol·g^−1^. However, when the initial polymer concentration increased, the removal efficiencies for the metals Co^2+^, Cr^3+^, Ni^2+^ and Zn^2+^ also increased (Table 4). For instance, with a polymer concentration of 10 g·L^−1^ (instead of 2 g·L^−1^ in Figure 5), retention reached 85%, 39%, 45% and 69% for Al^3+^, Co^2+^, Ni^2+^ and Zn^2+^, respectively. At the same time, the retention of the other inorganic elements also increased (57%, 71%, 10%, 35%, 73% and 54% for Ca^2+^, Fe^3+^, K^+^, Mg^2+^, Mn^2+^ and Sr^2+^, respectively).

**Table 4:** Removal efficiency of inorganic elements (%) in DW according to polymer concentration.

		removal efficiency (%)										

polymer concentration (g·L^−1^)	Al^3+^	Co^2+^	Cr^3+^	Ni^2+^	Zn^2+^	Ca^2+^	Fe^3+^	K^+^	Mg^2+^	Mn^2+^	Sr^2+^	pH

5	81	27	>98	37	62	34	63	44	4	0	32	5
10	85	39	>98	47	69	57	71	42	9	35	56	5
15	88	46	>98	42	74	74	69	40	13	55	72	5
20	88	52	>98	47	74	86	70	36	22	72	84	5

### Detailed analysis of the discharge water after treatment with polyBTCA-CD

Among the 189 substances/parameters analyzed in the raw DW, 35 were detected: sixteen inorganic elements including twelve metals, and seven APs (Table 5). The treatment by activated polyBTCA-CD affected all water parameters and all substances detected in the raw DW except for Na. As in previous experiments on DWs, it was observed that the metals were not efficiently adsorbed by the polymer with a removal efficiency reaching 24%, 12%, 17% and 44% for Al^3+^, Co^2+^, Ni^2+^ and Zn^2+^, respectively. Unlike metals, it appears that most of the organics were retained with removal efficiencies higher than 50%; for example 4tOP, 4nNP, monoethoxylate nonylphenol and monoethoxylate octylphenol were efficiently removed (81, 66, 83 and 77%, respectively). Moreover, some substances were retained in large quantities on the polyBTCA-CD such as chloride (130 mg·L^−1^) and calcium (119 mg·L^−1^), which is not reflected by the removal efficiency because of the large amounts present in the initial DW. The sodium concentration remained stable after adsorption due to the fact that the material was initially activated by NaHCO_3_.

**Table 5:** Extensive analysis on raw and treated DW by polyBTCA-CD (concentration = 2 g·L^−1^).

		initial concentration	final concentration	removal (%)

physicochemical parameters (mg·L^−1^)	pH	8	8	—
	BOD-5	60	56	7
	COD	847	325	62
	hydrocarbon index C10-C40	0.8	0.2	75
	total cyanide	0.39	0.35	10
	AOX	1.6	0.9	42
	nitrites	121	105	13
	Kjeldahl nitrogen	44	30	31
	total nitrogen	200	182	9

inorganic elements (mg·L^−1^)	chloride	3140	3010	4
	sulphate	208	201	3
	potassium	98	94	4
	calcium	787	668	15
	magnesium	2.3	2.1	9
	manganese	0.11	0.09	17
	sodium	1666	1667	—
	sulfur	91	83	9
	aluminum	0.09	0.07	24
	cobalt	2.82	2.49	12
	iron	0.37	0.27	27
	molybdenum	0.07	0.06	9
	nickel	0.34	0.28	17
	selenium	0.1	0.09	10
	strontium	0.27	0.26	7
	zinc	0.9	0.5	44

organic substances (µg·L^−1^)	1,2-dichloroethane	1.2	0.7	42
	chloroform	4.4	3.2	27
	dichlorobromomethane	0.8	<0.5	>38
	4-*tert*-octylphenol	2.1	0.4	81
	4-nonylphenol	0.3	<0.1	>66
	monoethoxylate nonylphenol	5.62	0.97	83
	diethoxylate nonylphenol	2.05	0.96	53
	monoethoxylate octylphenol	177	41	77
	diethoxylate octylphenol	177	130	27
	4-*tert*-butylphenol	26	14	46

## Discussion

To explain the adsorption performance of polyBTCA-CD, a chemisorption mechanism involving several interactions can occur including ion exchange, electrostatic interactions, inclusion complexation and/or precipitation [3,12–16].

The polymer, without an activation step, removed 20% of the total metal load in spiked solutions (SS) containing five metals at 10 mg·L^−1^ each. This could be attributed to surface adsorption and diffusion into the polymer network. However, it was observed that an activation step in an aqueous solution of sodium bicarbonate was necessary to enhance metal retention, as also reported by Ducoroy et al. [17], allowing for a removal efficiency for metals higher than 99% for Co^2+^, Ni^2+^ and Zn^2+^, through both electrostatic interactions and ion exchange. Indeed, during the process of polymer synthesis, the cross-linking agent BTCA was used, this compound presents four carboxylic functions and a previous study [18] showed that only two of its carboxylic groups reacted with the CD units to form the polymer network, the two remaining ones being available to react with cations through chemisorption (electrostatic interactions and ion exchange). Moreover, this treatment only converted carboxylic groups into carboxylate without altering the amorphous structure of the polymer, as seen from the X-ray spectra.

Precipitation could also explain the removal efficiency enhancement observed with the activated polymer since it induced a strong pH increase. Indeed, for the solution of five metals at concentration of 1 mg·L^−1^ (SS2) treated with activated polyBTCA-CD, the pH increased from 4.4 to 7.3, probably due to the basic character of the polymer, the COOH groups of which have been converted into COO^−^Na^+^ groups inducing metal precipitation, notably for Al^3+^ and Cr^3+^.

With the highest concentration tested (10 mg·L^−1^, which corresponds to 5 mg of each metal per gram of polymer), the removal efficiency represents 0.466 mmol of metals adsorbed per gram of polymer, that is lower than the theoretical ion exchange capacity, estimated by titration to be 0.705 mmol of COOH functional groups per gram of polymer. In theory, in order to saturate the COOH functional groups, a concentration of 6.5 mg of each metal per gram of polymer (i.e., 13 mg·L^−1^) would have been necessary. In the DWs, focusing on Ca^2+^ only, 119 mg·L^−1^ were retained by the polymer, representing a concentration of 1.49 mmol·g^−1^ of material. Thus, the IEC of the polymer (0.705 mmol·g^−1^) was exceeded. This observation confirmed the fact that the inorganic elements were not only retained by ion exchange and/or electrostatic interactions, but other interactions occurred, such as precipitation and/or physical phenomena (surface adsorption, diffusion into the network and/or hydrogen bonding).

This study demonstrated the efficiency of activated polyBTCA-CD to treat the inorganic load, but also to treat the organic pollutants. It can be noted that the analysis of 26 substances present in the DW tested, showed that the polymer is able to adsorb 4-*tert*-butylphenol, 1,2-dichloroethane and chloroform, but also APs and the organo-halogenated compounds (represented by the AOX parameter). For the retention of APs, no difference was noted between 4NP, 4nNP and 4tOP. Heavy PAHs were better retained than light ones. This observation could be partially explained by the greater hydrophobicity of heavy PAHs compared with light ones [19]. Indeed, in the case of interactions with organics, CD units play an important role. The hydrophobic cavities of these molecules allow for the formation of inclusion complexes with PAH guest molecules (organic substances) [3,20]. If the guest compounds present an appropriate molecular size and structure to enter into the CD cavity the more hydrophobic the organic substances, the more stable the inclusion complexes.

The material was also able to efficiently treat complex solutions containing metals, PAHs, APs and other substances. However, although in spiked solutions no competition effect was revealed between substance groups, these phenomena appeared in more complex solution such as DWs. Indeed, the retention of inorganic elements decreased from an average of 87% for the five metals in SS to 26% in DWs. It appears that other elements present in DW were retained by the polymer, for instance Fe^3+^, Mn^2+^, Mg^2+^ and Ca^2+^. Discharge waters from ST industries are complex matrices containing not only metals, PAHs or APs but also other elements including anions and salts, sometimes exceeding one gram per liter [21]. In this study, calcium and chlorides were present in the DWs at concentrations of 500 and 3 000 mg·L^−1^, respectively. In fact, a large amount of these other inorganic elements could interact with the polymer, notably Ca^2+^, which was retained at 119 mg·L^−1^. Thus, the low retention of target metals (Al^3+^, Co^2+^, Cr^3+^, Ni^2+^ and Zn^2+^) by the adsorbent could be due to the simultaneous presence of high quantities of K^+^, Na^+^, Mg^2+^ or Ca^2+^, which can saturate the carboxylate functions of the polymer and compete with the target metals for access to the active sites [22–25]. These observations were confirmed by the tests conducted in SS in the presence of calcium. An increase of polymer concentration in DWs (10 g·L^−1^ instead of 2 g·L^−1^) yielded better results in terms of removal efficiency for all metals. However, while the retention of Co^2+^, Ni^2+^ and Zn^2+^ was reduced in the presence of Ca^2+^, this was not always the case for Al^3+^ and Cr^3+^. In some cases, Al^3+^ and Cr^3+^ exhibited unexpected behavior.

In solutions at low concentrations (SS2, SS3 and SS4), Al^3+^ and Cr^3+^ were removed with low efficiency compared to other metals, but in solutions at higher concentrations (SS1) or in the presence of calcium (SS5), an increase of removal efficiency was observed whereas for the three other metals, retention decreased. In the case of mixtures, the same observations were made, Al^3+^ and Cr^3+^ removal being systematically lower, whereas for the three other metals the removal efficiency remained as high as 99%. Moreover, in DWs, Al^3+^ was retained by an average of 60% while the others did not exceed a removal efficiency of 30%. No simple explanation could clarify these phenomena but some specific characteristics of these two metals must be pointed out. Indeed, the behavior of both Al^3+^ and Cr^3+^ is highly pH dependent, with complex speciation patterns [26–28]. Since the pH value was not kept constant during tests, the pH variations probably led to changes in speciation of the two metals, leading to this unexpected behavior compared to the other metals. Moreover, it can be noted that Al^3+^ and Cr^3+^ have the smallest ionic radii of the five metals and a low electronegativity, which are two parameters that decrease metal removal as described by Al-Rub and co-workers [29].

## Conclusion

Throughout this study, it was demonstrated that polyBTCA-CD is a versatile sorbent able to retain substances present at concentrations close to a few milligrams per liter (metals and other inorganic elements) but also at trace concentrations (µg·L^−1^ for organics). Although ion exchange on the one hand, and host–guest inclusion on the other hand were the main phenomena interacting between adsorbent and solutes, the interpretation of the results was made difficult due to the wide diversity of polluting species present in DWs, involving numerous other mechanisms in the adsorption (classified for instance as chemisorption and physisorption phenomena) [3,12,20]. Thus, further studies are needed to better understand how water parameters impact the different routes of pollutant retention, which is of great interest for future applications of non-conventional adsorbents in industry.

## Experimental

### Synthesis and characterization of the material

The cross-linked polymer (Figure 1) was prepared in a single step by crosslinking hydroxypropyl-β-cyclodextrin (HPβCD; Kleptose HPB^®^, DS = 0.62, Roquette, Lestrem, France) using 1,2,3,4-butanetetracarboxylic acid (BTCA; Aldrich, Milwaukee, WI). The synthesis procedure has already been described in detail by Martel et al. [10,30]. Based from their methodology, the typical cross-linking reaction was carried out as follows: in a reactor, 0.37 mol·L^−1^ of sodium dihydrogenophosphate (Na_2_H_2_PO_2_·H_2_O, catalyst), 0.85 mol·L^−1^ of BTCA and 0.18 mol·L^−1^ of HPβCD were dissolved; the resulting solution was then concentrated by evaporation, and the mixture heated to 140 °C. These last two steps were performed under vacuum in a rotary evaporator; the polymer formed was re-suspended through addition of water to the reactor; the polymer was then filtered and purified by several washings with water. It was dried at 50 °C until constant weight, yielding a white powder. The yield of the reaction was equal to 87.6 %.

To activate the polyBTCA-CD by converting the carboxylic functions to their carboxylate form, the material was stirred for 4 h in an aqueous solution of 4 g·L^−1^ NaHCO_3_ (saturated) then extensively washed with osmosed water to remove unreacted reagents, and finally oven dried at 50 °C.

The ion exchange capacity (IEC) of the material was determined by pH-metric titration according to the calcium acetate method [31]. A solution of calcium acetate (2% w/w) was prepared in osmosed water. A weighed sample of dry polymer was stirred into the calcium acetate solution for 2 h following calibration with oxalic acid. Then, the solution was filtered, and the amount of acetic acid formed was measured by titration using a sodium hydroxide solution (0.05 M). The amount of ion exchange functions was equal to the amount of acetic acid present in solution. The results were expressed in mmol of COOH functional groups per gram of polymer.

The point of zero charge (PZC) values of both non-activated and activated polyBTCA-CD were determined by titration with the salt addition method. 50 mL of 0.1 M NaCl solution was placed in each of nine beakers. The pH of each solution was adjusted from 3 to 11 with one pH unit increment using a pH meter (pH meter, model 3110, WTW, Alès, France) with aqueous 0.1 M NaOH and 0.1 M HCl. Then 0.15 g of polymer was added to each beaker. The solutions were stirred for 48 h to reach equilibrium then the resulting pH (pHf) was measured. The difference between the initial and final pH values (ΔpH) was plotted against the initial pH. The PZC was represented by the point ΔpH = 0 [32].

X-ray diffraction analysis (XRD) was performed on a Bruker D8 Advance diffractometer using Cu Kα radiation with a wavelength of λ = 0.15406 nm produced at 40 kV and 40 mA. XRD data were collected over the 2θ range from 10° to 60° at every 0.02° with a scan speed of 0.5 s per step. For polymer, the diffraction profiles are divided into 2 parts: peaks related to diffraction of crystallites and a broad peak related to scattering of the amorphous phase. The assumption is that the areas are proportional to the scattering intensities of crystalline and amorphous phases. Thus, the percentage of polymer that is crystalline was determined from Equation 1 [33]:

[1]
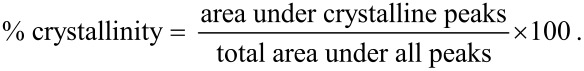


The polymer was also characterized by solid-state ^13^C NMR techniques such as cross polarization magic angle spinning (CP MAS) and MAS. The spectra were recorded with a Bruker spectrometer operating at 75.47 MHz and 303 K. The compounds were placed in a zirconium rotor, 4 mm in diameter and 21 mm high. The chemical shifts were recorded relative to tetramethylsilane with benzene as secondary reference. The Hartmann–Hahn condition was satisfied during cross polarization magic angle with 1.5 ms of contact time under the following conditions: repetition time 8 s, 1H90° pulse length 4 µs, and spin rate at 10 kHz.

### Adsorption tests

To determine the ability of the material to treat inorganic and organic load, several batch experiments were carried out with two kinds of solutions: spiked solutions (SS) containing different substances at several concentrations that are typical of ST discharge waters (DWs) [34] and real ST industrial DWs themselves.

SS contained metal sulfate salts (Al_2_(SO_4_)_3_·16H_2_O; CoSO_4_·7H_2_O; CrK(SO_4_)_2_·12H_2_O; NiSO_4_·6H_2_O; ZnSO_4_·7H_2_O) purchased from Aldrich and used without further purification. The sixteen PAHs of the US EPA list [35] and three APs were purchased from Supelco Sigma Aldrich (Saint-Quentin Fallavier, France) and used as received; eight light PAHs: naphthalene (NAP), acenaphthene (ACE), acenaphthylene (ACY), fluorene (FLU), phenanthrene (PHE), anthracene (ANT), fluoranthene (FLT) and pyrene (PYR); eight heavy PAHs: benz[*a*]anthracene (BaANT), chrysene (CHY), benzo[*b*]fluoranthene (BbFLT), benzo[*k*]fluoranthene (BkFLT), benzo[*a*]pyrene (BaPYR), dibenz[*a*,*h*]anthracene (dBahANT), indeno[1,2,3-*cd*]pyrene (IcdPYR) and benzo[*g*,*h*,*i*]perylene (BghiPL); three APs: 4-nonylphenol (4NP, CAS no. 84852-15-3), 4-*n*-nonylphenol (4nNP, CAS no. 104-40-5) and 4-*tert*-octylphenol (4tOP, CAS no. 140-66-9). Calcium chloride and sodium bicarbonate were purchased from Fischer Scientific (Illkirch, France) and used as received. Each SS was prepared from stock solutions in osmosed water.

#### Metal adsorption

In order to examine the effect of NaHCO_3_ treatment (activation), the adsorption capacities of the non-activated and the activated polymer polyBTCA-CD were compared with a solution containing the five metals (Al^3+^, Co^2+^, Cr^3+^, Ni^2+^ and Zn^2+^) each at a concentration of 10 mg·L^−1^. A kinetic study was also performed to determine the appropriate contact time between the material and the polymetallic solutions at concentrations of 1 and 10 mg·L^−1^ for each metal. Then, metal adsorption was determined by several polymetallic solutions containing the five metals described previously. Different concentrations of metals were tested: 10 mg·L^−1^ (SS1) and 1 mg·L^−1^ (SS2) for each metal and two solutions reproducing DW concentrations (SS3 and SS4). Moreover, two experiments were performed with metals in the absence (SS4) or presence (SS5) of CaCl_2_ at DW concentrations, in order to observe the influence of salt concentrations (Table 6).

**Table 6:** Concentrations of metals and calcium expressed in mg·L^−1^ in the spiked solutions.

	Al^3+^	Co^2+^	Cr^3+^	Ni^2+^	Zn^2+^	Ca^2+^	pHi

SS1	9.12	9.71	10.05	9.46	9.37	<0.5	3.9
SS2	0.91	0.97	1.01	0.95	0.94	<0.5	4.4
SS3	0.95	0.76	0.015	0.10	0.36	<0.5	4.8
SS4	2.17	1.34	0.038	0.20	0.76	<0.5	4.8
SS5	2.26	1.33	0.035	0.20	0.75	457	4.7

#### PAH and AP adsorption

To determine the PAH adsorption, solutions were prepared with the sixteen PAHs: eight were considered as light PAHs, i.e., with a lower molecular weight, (NAP, ACY, ACE, FLU, PHE, ANT, FLT, PYR) and eight as heavy PAHs (BaANT, CHY, BbFLT, BkFLT, BaPYR, IcdPYR, dBahANT, BghiPL). Two PAH concentrations were tested: 3229 and 7243 ng·L^−1^ for the sum of the sixteen PAHs, i.e., for the first an average concentration of 1543 ± 261 ng·L^−1^ and 1686 ± 410 ng·L^−1^ (SS6; pHi 6), and for the second an average of 3508 ± 454 ng·L^−1^ and 3735 ± 333 ng·L^−1^ (SS7; pHi 6) for the sum of the light and the heavy PAHs, respectively. In the case of the APs, 4NP, 4nNP and 4tOP were used to prepare solutions at two concentrations: SS8 (pHi 6) containing 44, 53 and 59 µg·L^−1^ and SS9 (pHi 6) containing 662, 720 and 889 µg·L^−1^ 4NP, 4nNP and 4tOP, respectively.

#### Metals and organic adsorption in mixtures

To study adsorption capacities when substances are in mixture, seven solutions were prepared: mixture of five metals (pHi 4.4), mixture of sixteen PAHs (pHi 6), mixture of three APs (pHi 6), mixture of five metals + 16 PAHs (pHi 4.4), mixture of five metals + three APs (pHi 4.4), mixture of 16 PAHs + three APs (pHi 6.1) and mixture of five metals + 16 PAHs + three APs (pHi 4.4). In each solution, the average concentration was 1.08 ± 0.11 mg·L^−1^ for each metal, 365 ± 29 ng·L^−1^ for each light PAH, 419 ± 24 ng·L^−1^ for each heavy PAH and 53 ± 24 µg·L^−1^ for each AP.

#### Adsorption capacities in discharge waters

Five samples of real discharge waters (DWs) were collected from Zindel Industry located in Seloncourt (Doubs, France) which is specialized in chemical coatings and any processes for the corrosion protection of metal parts intended for the automotive and building sectors. Their process waters mainly contain metallic pollutants (e.g., Cr^3+^, Ni^2+^ and Zn^2+^) coming from rinsing and washing baths. Following the tests conducted on SS, metal retention capacity was also tested on five different industrial DWs. In one DW, the effect of polymer dose was tested: 5, 10, 15 and 20 g·L^−1^. Moreover, one more detailed analysis was performed in order to see if the polymer can retain other substances present in the DW.

### Batch experiments

In each experiment, 2 g·L^−1^ of material was stirred (250 rpm) with a fixed volume of polluted solution (with no modification of the initial pH value) at room temperature for 4 h. After treatment, the solutions were left to settle for 1 h, and the supernatant was analyzed. Chemical analyses were performed in the initial and in the treated solutions and the results expressed in concentration and removal efficiency. Under the same conditions, a control experiment was performed without pollutants in order to check whether pH variations occurred in non-activated and activated polyBTCA-CD.

### Chemical analyses

For each SS or DW, initial and final pH values were measured. Metal concentrations were determined by inductively coupled plasma atomic emission spectroscopy (ThermoFisher, iCAP 6500 radial model, Courtaboeuf, France) after a step of acid digestion for DWs, following a previously reported method [21]. The analysis of the sixteen PAHs was performed by liquid–liquid extraction with hexane followed by separation and detection on a system composed of a GC apparatus and a triple quadrupole spectrometer (GC-MS/MS, Agilent, Massy, France) according to a method described in detail by Crini and co-workers [36]. Three APs (4NP, 4nNP, 4tOP) were analyzed by a certified laboratory (CARSO LSEHL, Lyon, France), by liquid–liquid extraction followed by separation and detection on GC-MS/MS according to the standard NF EN ISO 18857-1. For the detailed analysis, 189 substances and 17 water parameters were analyzed, before and after treatment of the DW, by a certified laboratory (CARSO LSEHL, Lyon, France).
